# Special Issue “Primate Phylogeny and Genetics”

**DOI:** 10.3390/genes15010068

**Published:** 2024-01-03

**Authors:** Ute Radespiel, Christian Roos

**Affiliations:** 1 Institute of Zoology, University of Veterinary Medicine Hannover, Foundation, Buenteweg 17, 30559 Hannover, Germany; 2 Gene Bank of Primates and Primate Genetics Laboratory, German Primate Center, Leibniz Institute for Primate Research, 37077 Göttingen, Germany

New phylogenetic tools and population genetics methods have been developed and vastly advanced over the last decade. For example, they allow to delimitate cryptic species more accurately [1], to infer species trees under incomplete lineage sorting and hybridization [2,3], to model alternative demographic population histories [4], to identify barriers to gene flow in various types of landscape [5], and provide detailed insights into the genetic diversity of a given taxon [6]. In this Special Issue (SI), “Primate Phylogeny and Genetics”, ten author teams assemble contributions that report on new molecular findings or review recent advances in primate taxa from most major primate clades (Lemuriformes, Lorisiformes, Platyrrhini, Catarrhini) that occur in three continents (South America, Africa, Asia). While five papers focused on understudied phylogenetic patterns and processes in their study genus (*Eulemur*, *Propithecus*, *Nycticebus* and *Xanthonycticebus*, *Sapajus*, *Aotus*; contributions 1–5), a landscape genetics study of *Varecia variegata* (contribution 6), a phylogeographic study of *Gorilla gorilla diehli* (contribution 7), a population genomic study of *Callithrix jacchus* (contribution 8), a gut microbiome study comparing folivorous, frugivorous and omnivorous primates (contribution 9), and an article reviewing our current knowledge about *Rhinopithecus* genetics and genomics (contribution 10) complement and broaden the view on recent advances in this field. The main insights from the contributions to this SI are summarized below.

True lemurs (genus *Eulemur*) with 12 species are a good model to study how species boundaries can be maintained in the face of high levels of gene flow as they represent a recent radiation with at least five active hybrid zones. Everson et al. (contribution 1) applied several phylogenomic approaches to study whether ancient gene flow and reticulation may have shape the diversification history within this lemur genus. The authors found evidence for non-monophyletic relationships and reticulations, suggesting that hybridization was a prominent feature during *Eulemur* evolution from the past to the present.

Hawkins et al. (contribution 2) performed a comprehensive phylogenomic analysis with 200 sifaka (genus *Propithecus*) samples that were collected from all nine nominal sifaka species across a wide geographic range in Madagascar. Ultraconserved elements (UCEs) and complete mitochondrial genomes were used to investigate the phylogenetic relationships within this clade, which initially diverged about 9 million years ago (Mya). While most species were monophyletic in all analyses, the authors also detected signals of a yet unknown cryptic divergence within *Propithecus diadema*, one case of mitochondrial introgression (between *P. diadema* and *Propithecus edwardsi*), and some discordance between phenotypic and molecular divergence, which might only be resolved by additional material from so far unsampled regions.

Blair et al. (contribution 3) investigated the evolutionary history of slow lorises (genera *Nycticebus* and *Xanthonycticebus*) using four mitochondrial markers—mainly generated from museum specimens. Concordant with previous analyses, *Xanthonycticebus* forms the sister genus to *Nycticebus,* and both separated about 11.3 Mya. In *Xanthonycticebus*, two major clades were detected, which refer to northern and southern populations, and which led the authors to divide pygmy slow lorises into two species (*Xanthonycticebus pygmaeus* and *Xanthonycticebus intermedius*). Likewise, several clades were observed within *Nycticebus*, but more work is needed before any taxonomic conclusions can be drawn. As slow lorises are often illegally traded, this study is of great importance as now tools are available to identify the species and geographic origin of confiscated slow lorises.

Robust capuchin monkeys (genus *Sapajus*) are widely distributed in South America. This genus contains eight species, but their taxonomic classification is disputed. Martins et al. (contribution 4) used ddRADseq data from a total of 171 robust capuchin monkey individuals representing all eight putative species. They revealed strong evidence for the clear distinction and species status of *Sapajus nigritus*, *Sapajus robustus,* and *Sapajus xanthosternos*. For the remaining species, the results were less conclusive, and several clades were obtained, which, however, do not agree with the morphology-based classification and distribution of species. Overall, the authors suggest a provisional classification of *Sapajus* into nine species but also highlight the need for additional studies.

Storer et al. (contribution 5) studied another group of South American primates, the night or owl monkeys (genus *Aotus*). They made use of insertion polymorphisms of *Alu* elements, a kind of retrotransposon, to resolve phylogenetic relationships within the genus. By investigating a total of 314 loci in five taxa, they found 159 insertion polymorphisms, with 21 of them grouping *Aotus nancymaae* with *Aotus azarae*. Moreover, they provided a set of more than 1350 locus-specific PCR assays, which could be used for *Aotus* species identification as well as for phylogenetic and population genetic studies of this genus in the future.

Mancini et al. (contribution 6) applied landscape genetics approaches to the black-and-white-ruffed lemur (*V. variegata*) in southeastern Madagascar to infer environmental constraints of dispersal for this arboreal rainforest species. The authors found that this species prefers to disperse locally via the least rugged terrain and within tall-canopied forests, while forest presence predicted long-distance dispersal and multigenerational gene flow best. Given the ongoing loss of forests and habitat modifications in Madagascar, this study has strong conservation implications, as it advocates for high-quality forests and forest connectivity to secure the long-term stability of lemur populations.

A conservation perspective is also a major focus of the phylogeographic approach that was taken by Alvarez-Estape et al. (contribution 7) after generating 41 new gorilla genomes; among these, 25 were from the critically endangered Cross River Gorilla (*G. g. diehli*), from single shed hairs collected from gorilla nests. This highly innovative and non-invasive approach allowed the authors to infer three population clusters and recent inbreeding that is probably due to their isolation from other fragmented populations. However, it also provided evidence for past gene flow and connectivity between two sites, which highlights the importance of conservation efforts to stabilize existing populations and to reestablish connectivity between the last subpopulations of this gorilla subspecies, which add up to only about 250–300 individuals in the wild.

The common marmoset (*C. jacchus*) is one of the most used nonhuman primate models in biomedical research. Harris et al. (contribution 8) generated a high-quality reference genome using long-read sequencing technology and resequencing data of another 84 individuals housed in diverse research centers. They identified a total of 19.1 million single nucleotide variants, 2.8 million short insertion/deletion variants, and 74,088 missense variants in protein-coding genes; some of the latter are known to have clinical relevance in humans. Overall, the authors provide an important and valuable genomic resource for this widely used model species, which could help to develop genetically engineered marmoset disease models.

The gut microbiome study by Zhang et al. (contribution 9) used a metatranscriptomic approach to compare dietary adaptations in the microbiome of one folivorous (*Rhinopithecus roxellana*), one omnivorous (*Macaca mulatta*), and one frugivorous primate species (*Nomascus annamensis*) that were collected from the Shennongija National Park (*R. roxellana*, *M. mulatta*) and the Nanning Zoo (*N. annamensis*) in China. Indeed, this study showed that the gut microbiome differed adaptively between primate species with different dietary regimes. Such a host–gut microbiome association forms the basis of a possible host-symbiont co-evolution, which may be an essential component of primate dietary adaptations.

Snub-nosed monkeys (genus *Rhinopithecus*) are endemic to China, Myanmar, and Vietnam. All five species have relatively small distributions and are classified as either endangered or critically endangered. Nonetheless, they are among the most studied primates in terms of genetics. In the review article by Kuang et al. (contribution 10), the authors give a comprehensive overview of recent advances in snub-nosed monkey genetics and genomics and how this information has expanded our knowledge about the phylogeny, phylogeography, population genetic structure, landscape genetics, demographic history, and the molecular mechanisms of adaptation to folivory and high altitudes in this primate genus.

The ten contributions to this SI cover a wide range of primate taxa, and different types of genetic material and molecular markers are applied. Overall, these studies largely expand our knowledge about the genetic diversity, evolutionary history, and ecology of the taxa under investigation. Moreover, and this may be equally relevant considering the ongoing loss of primate habitats, fragmentation, and climate change, most studies have strong conservation implications, not only because we understand better how and where extant lineages evolved but also how fragile or resilient different taxa may be in view of future challenges. This is particularly important for primates as over 65% of species are threatened by extinction, and 93% have declining populations [7]. On the other hand, as our closest relatives, nonhuman primates are widely used in biomedical research, and the data presented by Harris et al. (contribution 8) could help to improve and refine the use of common marmosets for such purposes.
